# Effect of photobiomodulation on pain control after clinical crown lengthening surgery

**DOI:** 10.34172/japid.2021.014

**Published:** 2021-10-27

**Authors:** Mehrnoosh Sadighi, Masoumeh Faramarzi, Ramtin Chitsazha, Mohammad Ghasemi Rad, Sina Ranjbar

**Affiliations:** ^1^Dental and Periodontal Research Center, Faculty of Dentistry, Tabriz University of Medical Sciences, Tabriz, Iran; ^2^Department of Periodontics, Faculty of Dentistry, Tabriz University of Medical Sciences, Tabriz, Iran; ^3^Private practice, Tabriz, Iran

**Keywords:** Clinical crown lengthening, pain, photobiomodulatione

## Abstract

**Background:**

Photobiomodulation is a novel technique to reduce pain following different surgeries and treatments. This study aimed to investigate the effect of photobiomodulation on pain control after clinical crown lengthening procedures.

**Methods:**

Twenty patients were included and randomly assigned to two groups in this single-blind randomized clinical trial. The patients had been referred to the Periodontics Department, Tabriz Faculty of Dentistry, for crown lengthening surgery. In the laser group, diode laser therapy with a wavelength of 860 nm and a power of 100 mW was applied immediately after the surgery on the surgery day and three and seven days after the surgery. In the control group, the laser was turned off, and passive radiation was applied to the area as the test group for 30 seconds per session in non-contact mode. The pain was assessed by a visual analog scale (VAS) questionnaire on the study timelines. Data were analyzed with SPSS 20 using ANOVA and post hoc Tukey tests.

**Results:**

Twenty patients were included in each study group, where the pain was relieved significantly over time. On the first (5.50±1.18) and seventh (1.8±0.42) days, the pain intensity was similar in the test and control groups. However, on the third day, the laser group (2.90±0.74) experienced a significantly lower pain intensity than the control group (4.0±0.67).

**Conclusion:**

Photobiomodulation relieved pain after clinical crown lengthening surgeries.

## Introduction

The crown lengthening surgery is a technique to expose more tooth surfaces for esthetic or reconstruction purposes through lowering the gingival or bone tissue apically.^1^ The indications of this surgery are subgingival decay or broad decay that shortens the tooth length, fractures, and premature eruption of the anatomical tooth crown. Various techniques such as gingivectomy, undisplaced flap with/without bone surgery, apical resected flap with/without bone resection, and forced eruption with/without fiberotomy have been proposed for crown lengthening procedures.^2-4^ Selecting the technique depends on various factors like esthetics, crown-to-root ratio, root morphology, furcation and tooth location, and reconstruction possibility.^5,6^ Crown lengthening procedures have become an essential part of aesthetics, as they are widely used to enhance the esthetics and restorations in the esthetic area.^7^

Compared to bone surgeries, pure mucogingival procedures result in more pain. The technical differences between the procedures with varying exposure levels of bone connective tissue can lead to differences in postoperative pain intensities. Wide surgeries are associated with more pain than less invasive surgeries.^3^ A part of the patient’s pain and discomfort is associated with his mental status before the surgery and expectations.^8^ This also depends on the surgery duration, which can intensify the patient’s stress and anxiety, leading to more severe pain.^9^

Photobiomodulation (PBM) has been applied in dentistry for various purposes, including wound treatment, aphthous stomatitis, mucositis, nerve regeneration, postherpetic neuralgia (PHN), synovitis, arthritis, temporomandibular joint pathologies, acute abscesses, peripheral granuloma, chronic orofacial pains, and bone regeneration. The analgesic effects of PBM are due to stimulating the synthesis of androgenic endorphins like beta-endorphin, reducing cytokines, and inflammatory enzymes, pain threshold shift, morphological changes in neurons, reduced mitochondrion membrane potential, and blocking the fast axon flow, leading to the neural pathway blockage. Its inflammatory impact occurs because of the increased activity of phagocytes, number and diameter of lymphatic vessels, and decreased vessel permeability and micro-capillaries blood circulation, and decreased edemas.^10^

Previous studies investigated the impact of PBM on the gingival regeneration after gingivectomy procedures, concluding that such laser therapy can enhance wound healing after gingivectomy.^11,12^ Concerning the importance of pain relief in crown lengthening surgery, this study investigated the impact of PBM on pain intensity after clinical crown lengthening procedures.

## Methods

This single-blind clinical trial was registered in the Iranian Center of Randomized Controlled Trials with an IRCT code of 20180114038364N1 and confirmed by the Ethics Committee of Tabriz University of Medical Sciences (IR.TBZMED.REC.1398.1299). Twenty patients referring to the Department of Periodontics, Tabriz Faculty of Dentistry, for crown lengthening surgery, who met the inclusion and exclusion criteria, were selected.

Patients aged 1860 with no systemic disease were randomly assigned to two groups (n=10). The flap technique was used for crown lengthening surgeries, and all the surgeries were performed by a skilled surgeon by observing all surgery principles. The patients were blinded to the intervention type.

The test group was treated by the photobiomodulation with GaAlAs laser (Wuhan Gigaa Optronics Technology, China) in non-contact mode with a wavelength of 860 nm, a power of 100 mW, and an energy density of 3 J/cm^2^ immediately after the surgery on the surgery day and three and seven days after surgery.^13^ For the control group, the laser was turned off, and passive irradiation was carried out on the given area. Patients in both groups were visited on the first, third, and seventh days. The laser irradiation duration was 30 seconds per session. All the laser therapy processes were carried out by meeting the laser safety guidelines.^14^

The patient’s pain status was assessed by the visual analog scale (VAS) questionnaire up to the seventh day after the surgery. Painkillers were prescribed for all the patients for one week (Gelofen, 400 mg Ibuprofen, Daana Pharma Co.).

## Results

In this study, 20 patients were studied in two groups: control and intervention. The frequency of males and females in the laser (70% female, 30% male) and control groups (60% female, 40% male) was the same. In addition, the mean age in the control group was 31.2±5.4, with no significant difference from the laser group.

**Figure 1:**
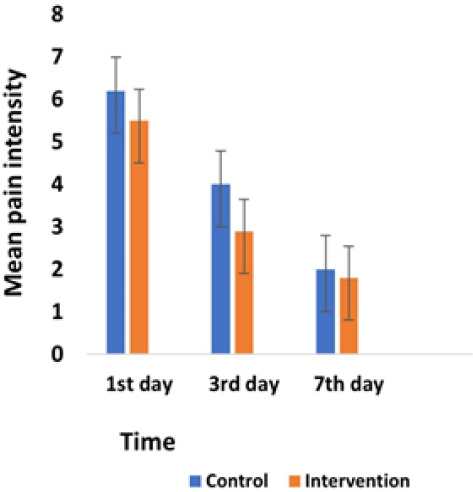
Comparison of the mean pain intensity between the patients in the control and intervention groups during the follow-ups.

The results showed that on the first day, pain intensity in both the control (6.2±0.79) and intervention (5.50±1.18) groups was similar. On the third day, pain intensity in the intervention group (2.90±0.74) was significantly lower than the control group (4.0±0.67). On the seventh day, the control group (2.0±0.67) experienced the same level of intensity as the laser group (1.8±0.42). Based on the Friedman test, the pain intensity decreased significantly over time in both groups (Figure 1).

**Figure 2:**
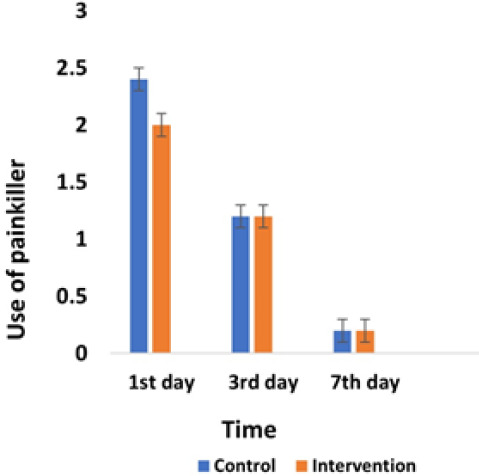
Comparison of the mean use of painkillers between the control and intervention groups during the follow-ups.

The mean use of painkillers on the first, third, and seventh days was the same in both the control and intervention groups, with no significant difference (Figure 2). According to the chi-squared test, no significant difference was observed in the frequency of painkiller use in both groups on the first, third, and seventh days.

## Discussion

In this study, pain intensity in both the control and laser groups had a significantly decreasing trend until the seventh day after surgery. It was also the same on the first and seventh days for both groups. However, on the third day, the pain intensity in the laser group was significantly lower than in the control group. The number of painkillers taken was similar in both groups. On the seventh day, 80% of patients in both groups took no painkillers.

In a study by Heidari et al,^15^ pain intensity in the periodontal flap surgery on the second, third, fourth, fifth, sixth, and seventh days after surgery was lower in the laser group. Furthermore, these patients took fewer painkillers during these days, consistent with the present study since we observed significant pain relief on the third day.

In a systematic review, Bjordal et al^16^ reported that the low-level laser could be effective in pain relief by reducing the biochemical markers, oxidative stress, and edema, which itself depends on the dose (effective dose range from 0.3 to 19 J/cm^2^ with a mean dose of 7.5 J/cm^2^). Furthermore, they reported that the analgesic impact of low-level laser is higher when it is irradiated with high density in the first 72 hours after surgery, which should continue with lower doses to accelerate regeneration.^15^ Studies by Lingamaneniet al^17^ and Kohale et al^18^ revealed that laser therapy could enhance wound healing after gingivectomy. Each researcher applied a different laser (InGaAsP) and obtained the same results.

Wound healing is a complicated and dynamic process with three overlapping phases. The first phase, or inflammatory phase, begins with tissue injury. The second phase, or the fibroblastic phase, includes the production of tropocollagen and collagen by fibroblasts. The final phase or remodeling lasts from months to years. In this stage, the irregular collagen fibers are removed and replaced by highly regular new fibers.^18,19^ Regarding the various stages of wound healing, photobiomodulation seems to be more effective in the fibroblastic phase of wound healing, including excessive fibroblast activity, angiogenesis, and epithelial proliferation. Effects of photobiomodulation on the fibroblast, including increased proliferation, increased growth factor secretion, and conversion into myofibroblasts, have already been observed in previous studies.^20^

Numerous studies have reported the impact of lasers on pain relief or wound regeneration. Carvalho et al^21^ reported pain relief by photobiomodulation in treating radiotherapy-induced mucositis in patients with maxillofacial cancers. Aras et al^22^ showed the impact of laser on reducing trismus and inflammation resulting from the impacted wisdom tooth surgery. Lioubavina-Hack et al^23^ presented a report on the effect of laser therapy on reducing the initial level of periodontal pathogens. Mechanisms of treatment by photobiomodulation are sophisticated, but they are necessarily dependent on the absorption of special visible red and near-to-infrared wavelengths in the photoreceptors of sub-cellular elements, particularly the electron transport chain (respiratory) in mitochondria. Photobiomodulation is a red or infrared light that stimulates the proliferation of fibroblasts through non-thermal effects. The possible mechanisms involved in pain relief are the stability of neural cell membrane, increased cell regeneration system, and increased ATP production. However, a study showed that this treatment method has no significant impact. In this regard, Heidari et al^13^ reported that laser photobiomodulation (PBM) has no impact on reducing free gingival graft (FGG). A low number of studied patients is among the reasons for differences in the results. Small size leads to the lack of reasonable certainty in the results. Age and gender are also among the variables affecting pain.

## Conclusions

Photobiomodulation (with GaAlAs laser) with a wavelength of 860 nm, a power of 100 mW, and an energy density of 3 J/cm^2^ immediately after surgery and on days 3 and 7 after surgery, as an adjunct, had limited effect on pain relief after clinical crown lengthening surgeries.

## Authors’ contributions

MF and MS planned the study. Data collection was carried out by SR. Statistical analyses and interpretation of data were carried out by RCH, MGH, and SR. The manuscript was prepared by RCH and MGH and revised by MS. All the authors have read and approved the final manuscript for submission.

## Ethics approval

The study protocol was approved by the Ethics Committee on Medical Research, Tabriz University of Medical Sciences.

## Competing interests

The authors declare that they have no competing interests regarding authorship and/or publication of this paper.
